# Association of serum potassium time in target range with cardiovascular outcomes in patients with HFpEF

**DOI:** 10.1136/openhrt-2025-003439

**Published:** 2025-08-21

**Authors:** Wenlong Xu, Zhiwen Xiao, Yating Tang, Yingxuan Li, Xingqiao Chen, Fengling He, Haoxiang Huang, Chuling Li, Yu Liu, Jiajun Zhou, Yuegang Wang, Jianping Bin, Yanmei Chen

**Affiliations:** 1 Department of Cardiology, Nanfang Hospital, Southern Medical University, Guangzhou, Guangdong, China; 2 Guangdong Provincial Key Laboratory of Cardiac Function and Microcirculation, Guangzhou, Guangdong, China; 3 Department of Cardiology, The Sixth Affiliated Hospital of South China University of Technology, Foshan, Guangdong, China

**Keywords:** Heart Failure, Education, Medical, Risk Factors

## Abstract

**Background:**

Serum potassium (sK) disorders are associated with worse outcomes in patients with heart failure with preserved ejection fraction (HFpEF). This study introduced a novel metric, time in target range (TTR), for long-term monitoring of sK levels and determined its prognostic value in patients with HFpEF from the Treatment of Preserved Cardiac Function Heart Failure with an Aldosterone Antagonist (TOPCAT) trial.

**Methods:**

The TTR for sK levels was defined as the percentage of time during which the sK was within the target range of 4.3–4.9 mmol/L, and was estimated using linear interpolation based on at least five valid measurements of sK. The association between sK TTR and cardiovascular (CV) events was estimated using adjusted Cox proportional hazards regression models.

**Results:**

A total of 3141 TOPCAT participants with HFpEF were evaluated over a median follow-up period of 3.9 years. A greater time within the range of 4.3–4.9 mmol/L for sK was associated with a lower risk of CV events in patients with HFpEF (HR: 0.712; 95% CI: 0.571 to 0.889). The benefits remained when the range of sK was set at 4.3–4.6, or 4.6–4.9, while no benefits or even negative effects were observed at 4.0–4.3, or 4.9–5.2 mmol/L. The association between a higher TTR and lower risk of CV outcomes was consistent across subgroups. The sK TTR predicted a lower risk of CV events, even after adjusting for traditional CV risk factors, mean sK and sK variability.

**Conclusion:**

Maintaining sK levels within the range of 4.3–4.9 mmol/L most of the time in patients with HFpEF is associated with a lower risk of CV events or all-cause mortality.

**Trial registration number:**

NCT00094302

**WHAT IS ALREADY KNOWN ON THIS TOPIC**

- Most studies reported a U-shaped association between serum potassium (sK) levels and cardiovascular (CV) events in patients with acute or chronic heart failure (HF), indicating that both hypokalaemia and hyperkalaemia were associated with poor outcomes in patients with HF.

**WHAT THIS STUDY ADDS**

- In this secondary analysis of the TOPCAT (Treatment of Preserved Cardiac Function Heart Failure with an Aldosterone Antagonist) trial, which included 3141 patients with HF with preserved ejection fraction (HFpEF) who underwent at least five repeated measurements of sK with a follow-up period of 3.9 years, we found that higher long-term sK time in target range (TTR) was associated with a decreased risk of CV events in patients with HFpEF, independent of mean sK and sK variability.

**HOW THIS STUDY MIGHT AFFECT RESEARCH, PRACTICE OR POLICY**

- This study reveals that management of sK should focus not only on achieving target range but also on maintaining most of the time achieved, and TTR may serve as an effective indicator for long-term potassium monitoring in CV risk assessment.

## Introduction

 Serum potassium (sK) disorders are relatively common in patients with heart failure (HF) because these patients usually have multiple comorbidities (eg, chronic kidney disease, older age and diabetes mellitus) and frequently receive diuretic therapy and renin–angiotensin–aldosterone system (RAAS) blockers.^1^ Disorders in sK levels can lead to changes in action potential and repolarisation time, resulting in alterations in the Q-T interval, ultimately causing cardiac abnormalities and increasing the risk of ventricular arrhythmias and death.^2^,^4^ Most studies reported a U-shaped association between sK levels and cardiovascular (CV) events in patients with acute or chronic HF, indicating that both hypo- and hyperkalaemia were associated with poor outcomes in patients with HF.^5^,^9^

As patients with HF with preserved ejection fraction (HFpEF) are often elderly with concomitant chronic kidney disease and other comorbid medical illnesses, they are at a particularly high risk for sK disorders during treatment.^10 11^ Recently, post hoc analyses of the Prospective Comparison of angiotensin receptor–neprilysin inhibitor (ARNI) with angiotensin-receptor blockers (ARB) Global Outcomes in HF with Preserved Ejection Fraction (PARAGON-HF) and Treatment of Preserved Cardiac Function Heart Failure with an Aldosterone Antagonist (TOPCAT) trials have shown that both hyperkalaemia and hypokalaemia are associated with an increased risk of CV events in patients with HFpEF.^12 13^ Another study found that the normal range of sK between 4.1 and 4.4 mEq/L is associated with a lower risk of CV and HF-related events in patients with HFpEF.^5^ However, these studies mainly focused on the prognostic implications of a single measurement using the initial or last recorded measurement, ignoring the potential implications of long-term sK disorders. A recent study indicated that greater variability is associated with higher rates of major adverse CV events and all-cause mortality in patients with HF.^14^ In addition, a single-centre observational study revealed that potassium dynamics were associated with substantial differences in mortality risk, indicating that the persistence of abnormal potassium levels was associated with a higher risk of death compared with that of patients who maintained or returned to normal levels.^15^ However, no metrics are available for long-term monitoring of sK within the target range, and no studies have focused on patients with HFpEF to examine the relationship between long-term sK control and outcomes.

Time in target range (TTR) is widely used to assess the stability of indicators such as blood pressure, haemoglobin A1c and body weight, reflecting variations over time both within and outside the target range.^16^,^18^ Many researchers, including ours, have applied TTR for population-level monitoring of blood pressure control and demonstrated that TTR could predict major adverse CV events independent of the mean achieved systolic blood pressure.^18^,^20^ As TTR can assess the degree of variability and reflect variations both within and outside the target range, sK TTR may be an effective assessment tool for evaluating long-term sK control and reflecting the longevity and consistency of blood potassium control. Accordingly, this study aimed to apply TTR to monitor long-term sK and determine the association between long-term sK TTR and the risk of CV outcomes in patients with HFpEF, using data from the TOPCAT trial.

## Methods

### TOPCAT trial design

The design, rationale and main results of the TOPCAT trial have been described previously (https://clinicaltrials.gov, NCT00094302).^19^ Briefly, the TOPCAT trial is an international, randomised, double-blind, placebo-controlled trial, at 233 sites in six countries (USA, Russia, Georgia, Canada, Brazil and Argentina), which was conducted from 10 August 2006 to 31 January 2012. This study included a total of 3445 participants, aged 50 years or older, with symptomatic HF and a left ventricular ejection fraction (LVEF) ≥45%, for a mean follow-up of 3.3 years. Eligible participants had a systolic blood pressure <140 mmHg, sK <5.0 mmol/L and either prior HF hospitalisation within 12 months or elevated natriuretic peptide levels (B-type natriuretic peptide ≥100 pg/mL or N-terminal pro-B-type natriuretic peptide ≥360 pg/mL). The key exclusion criteria were severe renal dysfunction (defined as an estimated glomerular filtration rate (eGFR) <30 mL/min/1.73 m^2^ or serum creatinine ≥2.4 mg/dL), severe systemic illness with a life expectancy of <3 years from randomisation and use of an aldosterone antagonist or potassium-sparing diuretic agent within 14 days before randomisation. Written informed consent was obtained from all the participants. The protocol was approved by the Institutional Review Board of each participating centre prior to the enrolment of the first participant.

### Analysed population

This study was a post hoc analysis of the data obtained from the TOPCAT trial. The TOPCAT Trial included patients with HFpEF and repeated measurements of sK levels, making it a suitable clinical trial for evaluating sK-TTR. Data are available from the National Heart, Lung and Blood Institute Biological Specimen and Data Repository Information Coordinating Center. For the present analysis, we excluded participants with an absence of follow-up sK data (n=9) or those with less than five sK measurements during follow-up (n=295). The final sample size for the analysis of TTR for sK and CV outcomes was 3141 participants (online supplemental figure S1).

### Assessments of mean sK, sK variability and sK TTR

sK levels were measured during screening and at each scheduled study visit, and the measurement was required within 1 week after a change in the study drug dose. The mean sK value was calculated by dividing the sum of all sK values by the total number of measurements. The variability in sK was represented by the SD calculated from all sK values. The sK TTR was defined as the percentage of time during which the sK was within the target range and was estimated using Rosendaal linear interpolation^21^ based on ≥3 valid measurements of sK during regularly scheduled visits before the occurrence of CV events between 2006 and 2012. To increase the reliability of data, we calculated TTR at least 5 times in TTR. The sK target range was determined as 4.3–4.9 mmol/L to match the current guideline and study-recommended target^15 22^ and determined as 4.0–4.3, 4.3–4.6, 4.6–4.9 and 4.9–5.2 mmol/L for additional analysis. Consequently, it was divided into three groups according to sK TTR tertiles (0–34.0%, 34.1–67.0%, 67.1–100%). A greater TTR (%) indicated that the participants achieved and maintained their target sK range for a longer duration within the follow-up period, before the occurrence of CV outcomes.

### Study outcomes

Follow-up visits to monitor symptoms, medications and events and to dispense the study drug were scheduled every 4 months during the first year of the study and every 6 months thereafter. The primary outcome of the present analysis was a composite of CV mortality, aborted cardiac arrest and hospitalisation for HF management, which was consistent with the primary endpoint of the TOPCAT trial. The secondary outcome of interest was the all-cause mortality. Only the first CV event was used in the analysis of participants who reached multiple endpoints. All occurrences of the individual components of the outcomes were adjudicated by a clinical endpoint committee at Brigham and Women’s Hospital according to the prespecified criteria.

### Statistical analysis

Continuous variables are expressed as mean±SD or median (percentile: 25–75). Categorical variables were presented as absolute numbers and percentages. In the analysis of the baseline data, missing values were filled using multiple interpolations, and the percentage of data with missing values was less than 5%. The TTR for sK was estimated using linear interpolation. The baseline characteristics were compared between the following sK TTR categories of tertile 1 (0–34.0%), tertile 2 (34.1–67.0%) and tertile 3 (67.1–100%) at screening, using analysis of variance or Kruskal-Wallis tests as appropriate, and categorical baseline data were compared using the χ^2^ test. The mean sK and sK variability associated with sK TTR was analysed using Pearson’s correlation analysis. We counted person-years from baseline to the date of death, loss to follow-up or 31 January 2012, whichever came first. The cumulative incidences of CV outcomes in the three sK TTR groups were estimated using the Kaplan-Meier method. The log-rank test was used to determine the survival distributions. The associations of mean sK, sK variability and sK TTR with the first occurrence of an efficacy outcome were estimated using HRs and 95% CIs derived from unadjusted and adjusted Cox proportional hazard models. We adjusted for baseline age, sex and body mass index (BMI) in Model 1, and further adjusted for race (white, black and others), region (Russia/Georgia, the USA, Argentina, Brazil and Canada (the Americas)), eGFR, LVEF, heart rate and medical history (hypertension, diabetes mellitus, CV disease (CVD)) in Model 2. We additionally adjusted for the use of beta blockers and diuretics in Model 3. Finally, we adjusted for mean sK and sK variability to determine whether sK TTR independently predicted CV outcomes. HRs were also estimated based on a 1-SD increase in the mean sK, sK variability and sK TTR. Differences in care and protocol adherence between the different centres in the TOPCAT trial may stem mainly from regional disparities. Therefore, we grouped participants by American versus non-American regions and examined the difference in TTR (%) between these regions. In the subgroup analyses, we performed correlation analyses for important variables, including sex, age, race, region, atrial fibrillation (AF), spironolactone use, BMI and eGFR to thoroughly validate the results. To assess the potential influence of the sK range on outcome, we performed a series of sensitivity analyses according to the narrower range of sK measurement (4.0–4.3, 4.3–4.6, 4.6–4.9 and 4.9–5.2) during follow-up. Statistical analyses were performed using the IBM SPSS Statistics V.27 programme (IBM, Armonk, New York, USA) and Stata V.17 software (Stata Corp, College Station, Texas, USA). Statistical significance was set at a two-sided p value of <0.05.

## Results

### Study population characteristics

A total of 3141 participants were included in this analysis; the mean age was 69 years, and 50.9% were women. The characteristics across the tertiles of baseline TTR are shown in table 1. Baseline demographics (age and sex), current smoking status, alcohol consumption, beta-blocker use, duretics use and heart rate were not significantly different among the three TTR groups at baseline. Compared with participants with the lowest TTR, participants in whom the highest TTR was performed during the follow-up were more frequently white (94.0% vs 85.9%) and Russian/Georgian (62.0% vs 39.4%), had higher eGFR (67.36 vs 64.88 mL/min/1.73 m^2^), with a lower percentage of New York Heart Association (NYHA) class 3–4 (28.6% vs 32.4%), had higher sK level (4.4 vs 4.1 mmol/L) and received angiotensin-converting enzyme inhibitors (ACEI)/angiotensin-receptor blockers (ARB) and spironolactone more frequently (85.8% vs 82.2% and 56.0% vs 39.5%). The classification and number of anti-HF medications used are shown in online supplemental tables S1 and S2.

**Table 1:** Baseline characteristics of participants according to serum potassium time in target range

Variables	Total (N=3141)	sK TTR group			P value
		0–34.0% (N=1047)	34.1–67.0% (N=1048)	67.1–100% (N=1046)	
Age (years)	69 (61–76)	68 (61–76)	69 (61–76)	69 (61–76)	0.983
Gender, n (%)					0.504
Male	1541 (49.1)	510 (48.7)	529 (50.5)	502 (48.0)	
Female	1600 (50.9)	537 (51.3)	519 (49.5)	544 (52.0)	
Race, n (%)					<0.001
White	2837 (90.3)	899 (85.9)	955 (91.1)	983 (94.0)	
Black	236 (7.5)	118 (11.3	72 (6.9)	46 (4.4)	
Other	68 (2.2)	30 (2.9)	21 (2.0)	17 (1.6)	
Regions, n (%)					<0.001
Americas	1546 (49.2)	634 (60.6)	515 (49.1)	397 (38.0)	
Russia/Georgia	1595 (50.8)	413 (39.4)	533 (50.9)	649 (62.0)	
BMI (kg/m^2^)	30.81 (27.14–35.54)	31.78 (27.76–36.80)	30.86 (27.40–35.68)	29.82 (26.49–34.29)	<0.001
eGFR (mL/min/1.73m^2^)	65.78 (53.96–79.32)	64.88 (52.98–78.69)	64.71 (53.48–78.47)	67.36 (56.13–80.42)	0.001
Blood pressure (mm Hg)					
Systolic	130 (120–140)	130 (120–139)	130 (120–138)	130 (120–140)	<0.001
Diastolic	80 (70–80)	78 (68–80)	80 (70–80)	80 (70–85)	<0.001
Heart rate (bpm)	68 (62–76)	68 (62–76)	68 (62–76)	68 (61–75)	0.339
Current smoker, n (%)	327 (10.4)	111 (10.6)	122 (11.7)	94 (9.0)	0.130
Alcohol drinking, n (%)					0.433
Non-drinker	2437 (77.7)	800 (76.5)	806 (77.1)	831 (79.4)	
Low-to-moderate drinker	652 (20.8)	226 (21.6)	224 (21.4)	202 (19.3)	
Heavy drinker	49 (1.6)	20 (1.9)	16 (1.5)	13 (1.2)	
Serum potassium					
Baseline (mmol/L)	4.3 (4.0–4.6)	4.1 (3.7–4.4)	4.4 (4.1–4.6)	4.4 (4.1–4.7)	<0.001
TTR (%)	52 (24–76)	13 (0.3–24)	52 (44–60)	85 (76–95)	0.000
Mean (mmol/L)	4.5 (4.2–4.7)	4.1 (3.9–4.3)	4.5 (4.3–4.7)	4.6 (4.5–4.7)	<0.001
SD (mmol/L)	0.3 (0.2–0.4)	0.3 (0.3–0.4)	0.4 (0.3–0.4)	0.3 (0.2–0.3)	< 0.001
LVEF (%)	56 (51–61)	57 (51–63)	56 (51–62)	55 (51–60)	0.005
NYHA class, n (%)					0.047
NYHA 1–2	2153 (68.6)	707 (67.6)	699 (66.7)	747 (71.4)	
NYHA 3–4	987 (31.4)	339 (32.4)	349 (33.3)	299 (28.6)	
Medical history, n (%)					
Hypertension	2869 (91.4)	932 (89.0)	965 (92.2)	972 (92.9)	0.003
Diabetes	985 (31.4)	345 (33.0)	349 (33.3)	291 (27.8)	0.010
CVD	2558 (81.5)	830 (79.3)	848 (81.0)	880 (84.1)	0.015
Dyslipidaemia	1880 (59.9)	628 (60.0)	679 (64.9)	573 (54.8)	<0.001
Atrial fibrillation	1105 (35.2)	402 (38.4)	402 (38.4)	301 (28.8)	<0.001
Paroxysmal atrial fibrillation	432 (39.1)	147 (14.0)	155 (14.8)	130 (12.4)	
Chronic atrial fibrillation	636 (57.6)	238 (22.7)	238 (22.7)	160 (15.3)	
Paroxysmal atrial fibrillation and chronic atrial fibrillation	30 (2.7)	13 (1.2)	7 (0.7)	10 (1.0)	
Unidentified	7 (0.6)	4 (0.4)	2 (0.2)	1 (0.1)	
Medication use, n (%)					
Spironolactone	1575 (50.1)	414 (39.5)	575 (54.9)	586 (56.0)	<0.001
Diuretics	2562 (81.6)	850 (81.2)	868 (82.9)	844 (80.7)	0.390
ACEI/ARB	2653 (84.5)	861 (82.2)	895 (85.5)	897 (85.8)	0.047
Beta-blocker	2441 (77.7)	820 (78.3)	813 (77.7)	808 (77.2)	0.837
Statin	1636 (52.1)	583 (55.7)	590 (56.4)	463 (44.3)	<0.001

Continuous variables are expressed as mean±SD or median (percentile: 25–75). Categorical variables are presented as absolute numbers and percentages.

ACEI, angiotensin-converting enzyme inhibitors; ARB, angiotensin-receptor blockers; ARNI, angiotensin receptor–neprilysin inhibitor; BMI, body mass index; CV, cardiovascular; CVD, cardiovascular disease; eGFR, estimated glomerular filtration rate; HF, heart failure; HFpEF, heart failure with preserved ejection fraction; HR, hazard ratio; LVEF, left ventricular ejection fraction; NYHA, New York Heart Association; PARAGON-HF, Prospective Comparison of angiotensin receptor–neprilysin inhibitor (ARNI) with angiotensin-receptor blockers (ARB) Global Outcomes in HF with Preserved Ejection Fraction; RASS, renin-angiotensin-aldosterone system; SD, standard deviation; sK, serum potassium; TOPCAT, Treatment of Preserved Cardiac Function Heart Failure with an Aldosterone Antagonist; TTR, time in target range.

### Mean sK, sK variability and sK TTR

As shown in table 1, the mean sK achieved for the entire study population was 4.5 mmol/L (IQR: 4.2–4.7 mmol/L). The sK variability was 0.3 mmol/L (IQR: 0.2–0.4 mmol/L). The overall sK-TTR was 52% (IQR: 24–76%). Participants in the highest sK TTR tertile had a higher mean sK and lower sK variability than those in the lowest tertile (4.6 (4.5–4.7) vs 4.1 (3.9–4.3) mmol/L, 0.3 (0.2–0.3) vs 0.3 (0.3–0.4) mmol/L, respectively; p<0.001). In the correlation analysis among TTR, mean sK and sK variability, sK TTR was moderately correlated with mean sK (R=0.302; p<0.001), and the associations between sK TTR and sK variability were weak or non-significant (R=−0.032; p=0.072) (online supplemental table S3). In addition, participants in the spironolactone group had a higher sK-TTR than those in the placebo group (57.0% vs 46.5%, p<0.001). The TTR was higher in the non-Americas than in the Americas (59.7% vs 43.7%, p<0.001). In the American population, the TTR was higher in the spironolactone group than in the placebo group (51.9% vs 32.4%, p<0.001), a phenomenon similar to that found in non-American populations (62.8% vs 57.0%, p=0.028) (online supplemental table S4).

### Associations of mean sK and sK variability with cardiovascular outcomes

As shown in table 2, in the unadjusted model, higher mean sK levels were associated with a lower risk for the primary outcome. However, in the fully adjusted model, the association between the highest tertile of mean sK in participants and the primary outcome (HR: 0.974; 95% CI: 0.789 to 1.203; p for trend=0.753), as well as the secondary outcome (HR: 0.953; 95% CI: 0.756 to 1.201; p for trend=0.645), was not significant compared with the lowest tertile. Similar results were obtained for the association between sK variability and CV outcomes. Additionally, the results were similar when the mean sK and sK variability were examined as continuous variables per 1-SD increase.

**Table 2:** HRs and 95% CIs of cardiovascular outcomes according to mean and variability for serum potassium

	Events (no.)	Follow-up duration (person-year)	Incident rate (per 1000 person-years)	Unadjusted HR (95% CI)	Model 1 HR (95% CI)	Model 2 HR (95% CI)	Model 3 HR (95% CI)
**Mean serum potassium**							
Primary outcome: death from cardiovascular causes, aborted cardiac arrest or hospitalisation for the management of heart failure							
Tertile 1	201	3283	61.23 (53.32–70.31)	1(ref.)	1(ref.)	1(ref.)	1(ref.)
Tertile 2	153	3639	42.04 (35.88–49.26)	0.694 (0.563 to 0.857)	0.719 (0.582 to 0.887)	0.829 (0.670 to 1.026)	0.830 (0.671 to 1.028)
Tertile 3	170	3567	47.67 (41.01–55.40)	0.783 (0.638 to 0.961)	0.812 (0.661 to 0.996)	0.960 (0.778 to 1.185)	0.974 (0.789 to 1.203)
P for trend				0.017	0.041	0.656	0.753
Per SD increase	524	10 489	49.96 (45.86–54.43)	0.928 (0.805 to 1.070)	0.927 (0.811 to 1.060)	0.982 (0.897 to 1.075)	0.985 (0.904 to 1.073)
Secondary outcome: all-cause mortality							
Tertile 1	165	3508	47.03 (40.38–54.78)	1(ref.)	1(ref.)	1(ref.)	1(ref.)
Tertile 2	129	3817	33.80 (28.44–40.16)	0.711 (0.565 to 0.896)	0.725 (0.576 to 1.067)	0.819 (0.649 to 1.035)	0.822 (0.650 to 1.038)
Tertile 3	143	3724	38.40 (32.60–45.24)	0.809 (0.647 to 1.012)	0.817 (0.653 to 1.023)	0.947 (0.752 to 1.193)	0.953 (0.756 to 1.201)
P for trend				0.060	0.073	0.611	0.645
Per SD increase	437	11 049	39.55 (36.01–43.44)	0.966 (0.852 to 1.095)	0.946 (0.836 to 1.071)	0.988 (0.905 to 1.079)	0.989 (0.908 to 1.077)
**Serum potassium SD**							
Primary outcome: death from cardiovascular causes, aborted cardiac arrest or hospitalisation for the management of heart failure							
Tertile 1	156	3183	49.01 (41.90–57.34)	1(ref.)	1(ref.)	1(ref.)	1(ref.)
Tertile 2	165	3654	45.15 (38.76–52.60)	0.931 (0.748 to 1.159)	0.941 (0.755 to 1.171)	0.937 (0.752 to 1.167)	0.938 (0.753 to 1.168)
Tertile 3	203	3652	55.59 (48.45–63.79)	1.146 (0.930 to 1.412)	1.140 (0.925 to 1.405)	1.030 (0.835 to 1.272)	1.024 (0.830 to 1.265)
P for trend				0.171	0.190	0.735	0.778
Per SD increase	524	10 489	49.96 (45.86–54.43)	0.976 (0.853 to 1.116)	0.958 (0.838 to 1.095)	0.964 (0.814 to 1.141)	0.962 (0.810 to 1.142)
Secondary outcome: all-cause mortality							
Tertile 1	125	3349	37.32 (31.32–44.47)	1(ref.)	1(ref.)	1(ref.)	1(ref.)
Tertile 2	134	3829	35.00 (29.55–41.45)	0.916 (0.717 to 1.169)	0.940 (0.736 to 1.199)	0.946 (0.741 to 1.209)	0.949 (0.743 to 1.213)
Tertile 3	178	3871	45.99 (39.70–53.26)	1.199 (0.953 to 1.507)	1.215 (0.966 to 1.528)	1.112 (0.882 to 1.402)	1.109 (0.879 to 1.399)
P for trend				0.090	0.074	0.322	0.336
Per SD increase	437	11 049	39.55 (36.01–43.44)	0.976 (0.848 to 1.124)	0.959 (0.837 to 1.099)	0.967 (0.824 to 1.135)	0.966 (0.822 to 1.135)

Primary outcome: death from cardiovascular causes, aborted cardiac arrest or hospitalisation for the management of heart failure; Secondary outcome: all-cause mortality.

Model 1, adjusted for baseline demographics (age and sex) and BMI.

Model 2, adjusted for Model 1 + region, race, eGFR, heart rate, New York Heart Association class, left ventricular ejection fraction, hypertension, diabetes mellitus and CVD.

Model 3, adjusted for Model 2 + use of beta-blockers and diuretics.

BMI, body mass index; CVD, cardiovascular disease; eGFR, estimated glomerular filtration rate; HR, hazard ratio; SD, standard deviation.

### Associations of sK TTR and cardiovascular outcomes

**Figure 1:**
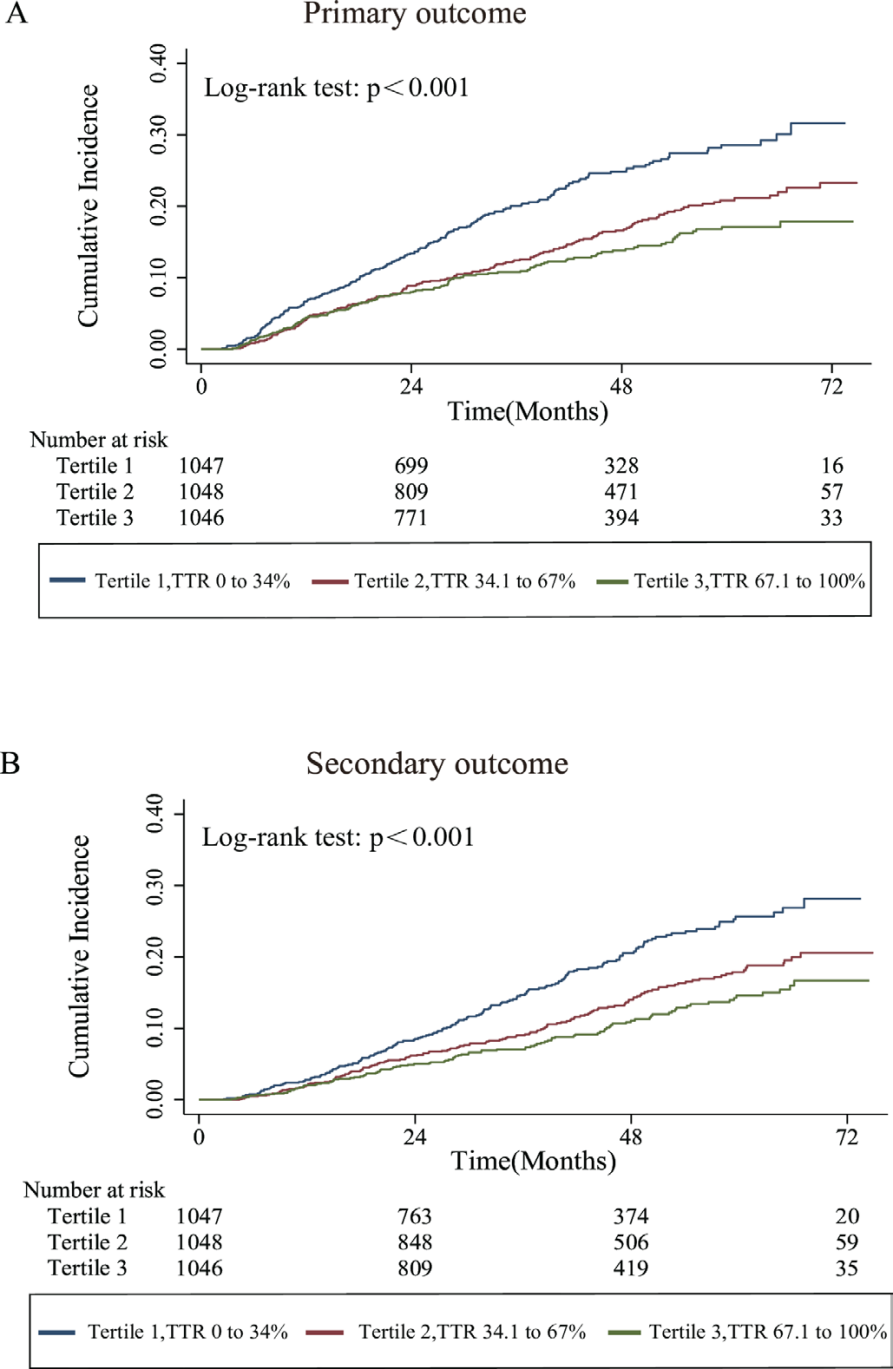
Kaplan-Meier curves for the tertiles of time in serum potassium target range and outcomes. The Kaplan-Meier survival function curves showed a lower cumulative incidence of outcomes in participants in the highest TTR tertile than in those in the lowest tertile. Primary outcome: death from cardiovascular causes, aborted cardiac arrest or hospitalisation for the management of heart failure; Secondary outcome: all-cause mortality. TTR, time in target range.

The Kaplan-Meier survival function curves showed a lower cumulative incidence of CV outcomes and all-cause mortality in participants in the highest TTR tertiles than in those in the lowest tertiles (all log-rank tests, p<0.05; figure 1). Cox proportional hazards models adjusted or unadjusted for covariates to estimate the association between TTR and CV outcomes are shown in table 3. In the unadjusted models, compared with the lowest TTR group, the highest TTR quartile group was associated with a decreased risk of the primary outcome, with HRs of 0.540 (95% CI: 0.435 to 0.670, p<0.001). Furthermore, after full adjustment, a higher sK TTR was significantly associated with a decreased risk of the primary outcome (HR, 0.712; 95% CI: 0.571 to 0.889; p=0.001). Similar results were obtained for the secondary outcomes. We converted the sK-TTR into a continuous variable to further verify the association between CV outcomes and sK-TTR. Each 1-SD increase in sK TTR was significantly associated with a decreased risk of the primary and secondary outcomes in the fully adjusted model, with HRs (95% CI) of 0.882 (0.804 to 0.968) and 0.847 (0.765 to 0.938), respectively (table 3). The association between a greater sK TTR and a decreased risk of CV outcomes remained significant after adjustment for mean sK or sK variability. When the sK TTR was converted to a continuous variable, for each 1-SD increase in TTR, the risk of the primary outcome decreased by 11.9% (HR, 0.881; 95% CI: 0.803 to 0.967) in Model 3 Plus sK SD For the secondary outcome, the risk was reduced by 15.4% (HR: 0.846; 95% CI: 0.765 to 0.937) with each 1-SD increase in TTR after full adjustment by Model 3 plus sK SD (table 4).

**Table 3:** HRs and 95% CIs of cardiovascular outcomes according to the tertile of time in target range for serum potassium

TTR	Events (no.)	Follow-up duration (person-year)	Incident rate (per 1000 person-years)	Unadjusted HR (95% CI)	Model 1 HR (95% CI)	Model 2 HR (95% CI)	Model 3 HR (95% CI)
Primary outcome: death from cardiovascular causes, aborted cardiac arrest or hospitalisation for the management of heart failure							
0–34.0%	221	3168	69.75 (61.14–79.58)	1(ref.)	1(ref.)	1(ref.)	1(ref.)
34.1–67.0%	171	3783	45.21 (38.92–52.52)	0.656 (0.537 to 0.802)	0.663 (0.543 to 0.810)	0.737 (0.602 to 0.901)	0.736 (0.601 to 0.900)
67.1–100%	131	3538	37.31 (31.46–44.25)	0.540 (0.435 to 0.670)	0.575 (0.463 to 0.714)	0.714 (0.572 to 0.891)	0.712 (0.571 to 0.889)
P for trend				<0.001	<0.001	0.002	0.001
Per SD increase	524	10 489	49.96 (45.86–54.43)	0.787 (0.720 to 0.860)	0.805 (0.736 to 0.881)	0.883 (0.805 to 0.969)	0.882 (0.804 to 0.968)
Secondary outcome: all-cause mortality							
0–34.0%	185	3423	54.04 (46.79–62.42)	1(ref.)	1(ref.)	1(ref.)	1(ref.)
34.1–67.0%	146	3941	37.04 (31.50–43.57)	0.671 (0.540 to 0.834)	0.692 (0.556 to 0.859)	0.742 (0.596 to 0.925)	0.744 (0.597 to 0.926)
67.1–100%	106	3685	28.77 (23.78–34.80)	0.527 (0.415 to 0.669)	0.543 (0.427 to 0.690)	0.645 (0.505 to 0.825)	0.645 (0.505 to 0.824)
P for trend				<0.001	<0.001	<0.001	<0.001
Per SD increase	437	11 049	39.55 (36.01–43.44)	0.780 (0.707 to 0.860)	0.788 (0.714 to 0.870)	0.847 (0.765 to 0.937)	0.847 (0.765 to 0.938)

Primary outcome: death from cardiovascular causes, aborted cardiac arrest or hospitalisation for the management of heart failure; Secondary outcome: all-cause mortality.

Model 1, adjusted for baseline demographics (age and sex) and BMI.

Model 2, adjusted for Model 1 + region, race, eGFR, heart rate, New York Heart Association class, left ventricular ejection fraction, hypertension, diabetes mellitus and CVD.

Model 3, adjusted for Model 2 + use of beta-blockers and diuretics.

BMI, body mass index; CVD, cardiovascular disease; eGFR, estimated glomerular filtration rate; HR, hazard ratio; SD, standard deviation; TTR, time in target range.

**Table 4:** Association of serum potassium time in target range and cardiovascular outcomes: further adjustment for mean serum potassium or serum potassium SD

Outcome	Fully adjusted+mean sK	Fully adjusted+sK SD		
	HR (95% CI)*	P value	HR (95% CI)†	P value
Primary outcome: death from cardiovascular causes, aborted cardiac arrest or hospitalisation for the management of heart failure				
0–34.0%	1(ref.)	0.002	1(ref.)	0.002
34.1–67.0%	0.733 (0.598 to 0.898)	0.003	0.735 (0.600 to 0.899)	0.003
67.1–100%	0.710 (0.568 to 0.887)	0.003	0.710 (0.569 to 0.886)	0.002
P for trend	0.001		0.001	
Per SD increase	0.881 (0.802 to 0.968)	0.008	0.881 (0.803 to 0.967)	0.008
Secondary outcome: all-cause mortality				
0–34.0%	1(ref.)	<0.001	1(ref.)	< 0.001
34.1–67.0%	0.741 (0.594 to 0.923)	0.008	0.742 (0.596 to 0.925)	0.008
67.1–100%	0.642 (0.502 to 0.822)	<0.001	0.643 (0.503 to 0.822)	< 0.001
P for trend	<0.001		<0.001	
Per SD increase	0.845 (0.762 to 0.936)	0.001	0.846 (0.765 to 0.937)	0.001

Primary outcome: death from cardiovascular causes, aborted cardiac arrest or hospitalisation for the management of heart failure; Secondary outcome: all-cause mortality.

Fully adjusted for baseline demographics (age and sex), BMI, region, race, eGFR, heart rate, New York Heart Association class, left ventricular ejection fraction, hypertension, diabetes mellitus, cardiovascular diseases and use of beta-blockers and diuretics.

* Fully adjusted+mean sK.

† Fully adjusted+sK SD.

BMI, body mass index; eGFR, estimated glomerular filtration rate; HR, hazard ratio; SD, standard deviation; sK, serum potassium.

### Sensitivity analyses

In subgroup analyses, we stratified the association between TTR and CV events by baseline age, region, sex, BMI, NYHA class, eGFR, AF, spironolactone use and race. The association between higher TTR and lower risk of CV outcomes was consistent across clinically important subgroups, such as age, region, sex, BMI, NYHA class, eGFR and AF (p>0.05) (online supplemental figures S2–S8). When stratified by spironolactone use and race, the HRs (95% CI) for all-cause mortality in the adjusted analyses were 0.740 (0.636 to 0.862) for participants using spironolactone and 0.963 (0.837 to 1.080) for those not using spironolactone (p=0.021) (online supplemental figure S9). We further investigated the association between sK TTR and CV outcomes stratified by spironolactone treatment, and the results did not reach a statistical difference (online supplemental table S5). Similar results were obtained for race (online supplemental figure S10). Consistently significant associations were observed in the multivariate Cox regression model adjusted for the number of anti-HF drugs (online supplemental table S6). To further validate the robustness of the results, we redefined the range of sK (4.0–4.3, 4.3–4.6, 4.6–4.9, 4.9–5.2) based on previous studies. In the redefined sK target ranges of 4.3–4.6 and 4.6–4.9 mmol/L, participants with higher TTRs were still associated with lower risks of CV outcomes, and the results were similar to the primary findings from the target range of 4.3–4.9 mmol/L (online supplemental tables S7 and S8). However, this association was not observed in the 4.0–4.3 and 4.9–5.2 mmol/L ranges (online supplemental tables S9 and S10). In a sensitivity analysis that restricted the analysis to individuals with more than three sK measurements, participants with higher TTRs were still associated with lower risks of primary and secondary outcomes (online supplemental table S11). Finally, in the unadjusted models, for the aborted cardiac arrest or ventricular tachycardia event, higher sK TTR was associated with a lower risk of aborted cardiac arrest or ventricular tachycardia outcome, whereas no correlation was found for mean sK and sK variability (online supplemental table S12).

## Discussion

This study is the first to use sK TTR to monitor long-term sK homeostasis in patients with HF. In this analysis of 3141 participants with HFpEF in the TOPCAT trial with a median follow-up period of 3.9 years, a greater time with an sK level within the target range of 4.3–4.9, or an even narrower range, predicted a lower risk of CV events and all-cause mortality. The association was independent of the traditional CV risk factors, mean sK and sK variability. This study suggests that maintaining normal potassium levels and greater TTR for sK in patients with HFpEF helps improve CV outcomes. Our study placed more emphasis on the duration of blood potassium in the target range, rather than on a particular point in time.

Given the high prevalence of abnormal sK levels in patients with HFpEF,^12 23 24^ there is a tremendous need for close monitoring of sK levels within therapeutic targets. Patients who actively maintain sK levels typically show significant improvements in CV outcomes, whereas those who do not maintain potassium levels face increased health risks, such as cardiac abnormalities and muscle weakness, which are attributed to unstable sK levels. TTR can consider sK variability along with the target range over time, representing a novel metric for monitoring long-term sK levels. In this study, we first introduced TTR to evaluate the prognostic implications of sK TTR for CV outcomes in patients with HFpEF and showed a linear association between TTR and the risk of composite CV events and all-cause mortality in patients with HFpEF.

Interestingly, this study showed that the patients in the spironolactone group had a higher sK TTR than those in the placebo group, suggesting that spironolactone use may be closely related to more stable sK homeostasis in patients with HFpEF, and it reduced the incidence of hyperkalaemia or hypokalaemia throughout the follow-up period. Nonetheless, in the overall TOPCAT population, spironolactone treatment did not show any benefit regarding the primary outcomes.^25^ The reduced risk of sK disorders with spironolactone did not translate into a reduced risk of CV events, perhaps partly because spironolactone might increase other events not caused by abnormal blood potassium, or because the patients in the spironolactone group had more comorbidities than those in the control group.^26^ In the subgroup analysis, we found that a higher TTR was associated with a significant reduction in all-cause mortality risk in the spironolactone group, suggesting that using the TTR as an assessment tool in clinical practice to adjust the dosage of spironolactone may potentially bring greater benefits in terms of overall mortality. Our findings suggest that spironolactone use among those adherent and responding to potassium, minimisation of hypokalaemic periods may confer benefits. Considering the heterogeneity of the population between Russia/Georgia and the Americas, we further analysed the relationship between TTR in regional subgroups and the long-term prognosis of HFpEF. We found that the association between TTR and CV outcomes remained consistent, indicating that heterogeneity between regions did not affect our robust results. We propose that the TTR could potentially serve as an effective indicator for long-term potassium monitoring in CV risk assessment. For patients with HF, especially those with chronic kidney disease or those treated with RAAS inhibitors, we suggest using the sK TTR for timely monitoring of potassium disturbances.

The sK target range used in this study was 4.3–4.9 mmol/L, in line with most normal sK ranges focusing on the HF population in most trials.^5 12 13^ The range is set from the median baseline potassium level of the participants (4.3 mmol/L) as the lower limit to the threshold for hyperkalaemia as the upper limit, and additional analyses are conducted in narrower ranges (4.0–4.3, 4.3–4.6, 4.6–4.9 and 4.9–5.2). The optimal range of sK levels in patients with HFpEF remains controversial. Traditionally, normal potassium levels are defined as 3.5–5.0 mmol/L. For patients with HFpEF, a post hoc analysis of the TOPCAT trial with randomised American patients showed that a sK level between 3.5–5.5 mmol/L was associated with a lower risk for mortality.^13^ However, increasing evidence supports an optimal potassium range that is narrower and at the higher end of what is considered the ‘normal’ range in patients with HF. Recently, a post hoc analysis of the PARAGON-HF trial showed that a sK level between 4–5 mmol/L was associated with lower rates of hospitalisation for HF than that observed in patients with a sK level <4 mmol/L or >5 mmol/L.^12^ Another retrospective, single-centre observational study found that a narrow sK interval of 4.1–4.4 mEq/L was associated with the best prognosis in patients with HFpEF.^5^ Our results showed significant benefits for CV outcomes at a higher TTR when the patients were within a sK range of 4.3–4.9, 4.3–4.6 or 4.6–4.9, but not significant or even negative effects with a sK target of 4.0–4.3 mmol/L or 4.9–5.2 mmol/L. These findings are consistent with those of most sK trials focusing on patients with HFpEF, and indicate that there is a narrower target range of sK than the normal range for patients with HFpEF. For patients with HFpEF, an sK range of 4.3–4.9 may be appropriate, and long-term maintenance within this range may offer optimal CV benefits.

A higher sK TTR reflects a lower variability and steadier sK levels during a specific period. Several possible mechanisms are involved in the high TTR and CV outcomes. First, potassium plays a crucial role in the maintenance of normal electrophysiological membrane potentials. A higher sK TTR may lead to a stable electrophysiological environment for cardiomyocytes, reducing the incidence of arrhythmia.^27^ Second, the long-term stability of sK tends to maintain vasodilatory contractile function, which may reduce blood pressure fluctuations.^28^ Finally, some studies have reported that sK fluctuations reflect a more severe presentation (as reflected metabolically or hormonally) with an increased rate of complications (eg, HF and renal failure); hence, sK fluctuations are a marker of severity.^29 30^ Many previous studies have demonstrated a U-curve relationship between sK and CV outcomes, indicating sK levels out of range on high versus low side were associated with poor prognosis in patients with chronic and acute HF.^5^,^9^ Our study demonstrated that sustained control of the sK within the target range could improve outcome. The lower TTR of sK may reflect HF decompensations, worsening of HF and dysregulations of comorbidities such as renal dysfunction. Conversely, a higher sK TTR could represent a marker of stability in patients with HFpEF and thus alleviate the underlying diseases or the need for lower use of medications. We emphasise the benefits of maintaining sK levels and recommend keeping sK within the range (4.3–4.9 mmol/L) as a key therapeutic goal. Although sK levels fluctuate naturally, they can be managed actively.

According to the frequency of potassium monitoring in the TOPCAT trial, we recommended the schedule for sK measurements as follows: first, the sK levels should be measured prior to initiating ACEI/ARB/diuretic therapy; then, they should be monitored again within 1 week of drug administration. For those patients with abnormal potassium levels, repeat measurement within 7 days was recommended. For those patients with stable potassium levels, it is recommended to monitor the potassium levels every 4 weeks and eventually extend to 6-month intervals. In cases involving medication adjustment, potassium monitoring should be carried out within 1 week of the adjustment. To effectively maintain sK levels, interventions for hyperkalaemia include dietary potassium restriction, reduction of RAAS inhibitor usage and the use of newer medications. Potassium binders or dialysis may be required for precise potassium control in specific patients such as those with renal impairment. Conversely, in cases of mild hypokalaemia in patients with HF, it is recommended to increase the dosage of ACEIs/ARBs/angiotensin receptor–neprilysin inhibitors (ARNIs) to meet guideline-recommended targets. In patients with persistent fluid overload and more severe hypokalaemia, loop diuretics are preferred to manage congestion, and potassium supplementation may also be considered. Further studies are warranted to determine whether aggressive management of dyskalaemia using potassium supplements for hypokalaemia or potassium binders for hyperkalaemia can reduce mortality in patients with HFpEF. The comprehensive application of these measures helps maintain potassium levels within the target range, ensuring long-term potassium stability and improving overall CV outcomes.

### Limitations

First, this was a post hoc analysis of the TOPCAT trial. The TOPCAT trial included participants exclusively from the USA, Russia and Georgia, and the ethnicity of more than 90% of the participants was white, so our findings may not be generalisable to patients with HFpEF in Asia and Africa. Further research with the inclusion of a more diverse population is warranted to determine the predictive value of TTR across all populations. Second, the study results may be influenced by certain factors, such as the potentially lower CV event rates among patients from Russia and Soviet Georgia and the possibility that some participants did not adhere to the study drugs. However, in the primary analysis, we adjusted for geographical and medication factors. Subgroup analyses based on ethnicity, geographical region and medication were conducted to validate the results. Although we were lenient on the inclusion criteria and performed sensitivity analyses and the results remained robust, this still did not completely avoid selection bias or survivorship bias from secondary analyses. Third, although the analyses were performed to adjust for baseline differences, other potential unobserved confounders may not have been considered, such as the use of antiarrhythmics, digoxin, magnesium level, variation of LV function, LV filling pressures and kidney function, which may confound the apparent association among sK TTR, mortality and CVD outcomes, and conclusions cannot be drawn about causality. Fourth, due to the lack of available data on sudden cardiac death outcome in the TOPCAT trial, we may further validate the association between sK TTR and sudden cardiac death outcome in a real-world HFpEF cohort in the future. Finally, the current study enrolled a predominantly HFpEF population, and further research including patients with a reduced ejection fraction is needed to determine the generalisability of sK-TTR in patients with HF with a full range of ejection fractions.

## Conclusions

sK TTR may be a useful metric for sK control in the HFpEF population. Maintaining sK levels within the therapeutic range of 4.3–4.9 mmol/L most of the time in patients with HFpEF was associated with a lower risk of CV events or all-cause mortality.

### Impact on daily practice

Maintaining higher long-term sK TTR was associated with a decreased risk of CV events in patients with HFpEF, independent of mean sK and sK variability.

## Supplementary material

10.1136/openhrt-2025-003439 online supplemental file 1

10.1136/openhrt-2025-003439 online supplemental file 2

## Data Availability

Data are available in a public, open access repository.
