# Supplementary material for: Association of serum potassium time in target range with cardiovascular outcomes in patients with HFpEF
Source: Open Heart. 2025 Aug 21;12(2):e003439. doi: 10.1136/openhrt-2025-003439 (PMC12410661; doi:10.1136/openhrt-2025-003439)
Supplement: online supplemental file 1 [file openhrt-12-2-s001.docx]

**Supplementary Online Content**

**Table S1. Classifications of Anti-Heart Failure Medication at Baseline**

**Table S2. The Numbers of Anti-heart failure Medication in the baseline**

**Table S3. Associations Between Serum Potassium Time in Target Range and Mean Serum Potassium or Serum Potassium Variability**

**Table S4. Serum Potassium Time in Target Range Differences in Placebo/ Spironolactone and Americas/non- Americas Group**

**Table S5.** **Hazard Ratios and 95% CIs of Cardiovascular Outcomes According to the Tertile of Time in Target Range for Serum Potassium stratify by Spironolactone Treatment**

**Table S6. Association of Time in Target Range for Serum Potassium with Cardiovascular Outcomes Adjusted for Number of Anti-Heart Failure Medication**

**Table S7. Sensitivity Analysis: Unadjusted and Adjusted Associations of Serum Potassium Time in Target Range (4.3–4.6) and Cardiovascular Outcomes**

**Table S8. Sensitivity Analysis: Unadjusted and Adjusted Associations of Serum Potassium Time in Target Range (4.6–4.9) and Cardiovascular Outcomes.**

**Table S9. Sensitivity Analysis: Unadjusted and Adjusted Associations of Serum Potassium Time in Target Range (4.0–4.3) and Cardiovascular Outcomes**

**Table S10. Sensitivity Analysis: Unadjusted and Adjusted Associations of Serum Potassium Time in Target Range (4.9–5.2) and Cardiovascular Outcomes**

**Table S11.** **Hazard Ratios and 95% CIs of Cardiovascular Outcomes According to the Tertile of Time in Target Range for Serum Potassium by Using ≥ 3 Serum Potassium Measurements**

**Table S12.** **Hazard Ratios and 95% CIs of Aborted Cardiac Arrest or Ventricular Tachycardia Event According to Mean, Variability and TTR for Serum Potassium**

**Figure S1. Selection of Study Population**

**Figure S2. Subgroup Analysis: The Association Between Serum Potassium Time in Target Range and Cardiovascular Outcomes According to Age**

**Figure S3. Subgroup Analysis: The Association Between Serum Potassium Time in Target Range and Cardiovascular Outcomes According to Region**

**Figure S4. Subgroup Analysis: The Association Between Serum Potassium Time in Target Range and Cardiovascular Outcomes According to Sex**

**Figure S5. Subgroup Analysis: The Association Between Serum Potassium Time in Target Range and Cardiovascular Outcomes According to BMI**

**Figure S6. Subgroup Analysis: The Association Between Serum Potassium Time in Target Range and Cardiovascular Outcomes According to NYHA class**

**Figure S7. Subgroup Analysis: The Association Between Serum Potassium Time in Target Range and Cardiovascular Outcomes According to eGFR**

**Figure S8. Subgroup Analysis: The Association Between Serum Potassium Time in Target Range and Cardiovascular Outcomes According to AF**

**Figure S9. Subgroup Analysis: The Association Between Serum Potassium Time in Target Range and Cardiovascular Outcomes According to the Use of Spironolactone**

**Figure S10. Subgroup Analysis: The Association Between Serum Potassium Time in Target Range and Cardiovascular Outcomes According to Race**

**Table S1. Classifications of Anti-Heart Failure Medication at Baseline**

|  |  | TTR group | | |  |
| --- | --- | --- | --- | --- | --- |
| Variables | **Total**  **(n=3141)** | **Tertile 1**  **(0% to 34.1%)**  **N = 1047** | **Tertile 2**  **(34.1% to 67.0%)**  **N = 1048** | **Tertile 3**  **(67.1% to 100%)**  **N = 1046** | **p Value** |
| Classifications of anti-heart failure medication, n (%) |  |  |  |  |  |
| Spironolactone | 1575(50.1) | 414(39.5) | 575(54.9) | 586(56.0) | ＜0.001 |
| ARB | 617(19.6) | 222(21.2) | 221(21.1) | 174(16.6) | 0.011 |
| ACEI | 2079(66.2) | 660(63.0) | 687(65.6) | 732(70.0) | 0.003 |
| Beta-blocker | 2441(77.7) | 820(78.3) | 813(77.7) | 808(77.2) | 0.837 |
| Diuretic | 2562(81.6) | 850(81.2) | 868(82.9) | 844(80.7) | 0.390 |

Abbreviations: ACEI, angiotensin-converting enzyme inhibitor; ARB, angiotensin receptor blocker.

**Table S2. The Numbers of Anti-heart failure Medication in the baseline**

|  |  | TTR group | | |  | |
| --- | --- | --- | --- | --- | --- | --- |
| Variables | **Total**  **(n=3140)** | **Tertile 1**  **(0% to 34.1%)**  **N = 1047** | **Tertile 2**  **(34.1% to 67.0%)**  **N = 1048** | **Tertile 3**  **(67.1% to 100%)**  **N = 1046** | | **p Value** |
| No. anti-heart failure medication, n (%) |  |  |  |  | | ＜0.001 |
| 0 | 21(0.7) | 7(0.7) | 7(0.7) | 7(0.7) | |  |
| 1 | 154(4.9) | 57(5.4) | 40(3.8) | 57(5.4) | |  |
| 2 | 682(21.7) | 270(25.8) | 213(20.3) | 199(19.0) | |  |
| 3 | 1397(44.5) | 489(46.7) | 460(43.9) | 448(42.8) | |  |
| 4 | 865(27.5) | 218(20.8) | 317(30.3) | 330(31.5) | |  |
| 5 | 21(0.7) | 6(0.6) | 10(1.0) | 5(0.5) | |  |

**Table S3. Associations Between Serum Potassium Time in Target Range and Mean Serum Potassium or Serum Potassium Variability**

| **Pearson Correlation** | **Serum Potassium Time in Target Range** | |
| --- | --- | --- |
|  | **R** | **p value** |
| **Mean serum potassium** | 0.302 | < 0.001 |
| **Serum potassium variability** | -0.032 | 0.072 |

**Table S4. Serum Potassium Time in Target Range Differences in Placebo/ Spironolactone and Americas/non- Americas Group**

| **Populations** | **TTR, %**  **median (interquartile range)** | |  | **TTR, %**  **median (interquartile range)** | | |
| --- | --- | --- | --- | --- | --- | --- |
|  | **Overall** | **p value** |  | **Placebo group** | **Spironolactone group** | **p value** |
| **Total** | 52.0(24.1-75.8) | **-** |  | 46.5(15.9-73.4) | 57.0(32.3-77.6) | < 0.001 |
| **Americas** | 43.7(16.2-68.0) | < 0.001 |  | 32.4(5.7-61.2) | 51.9(28.4-73.0) | < 0.001 |
| **non-Americas** | 59.7(33.2-81.8) |  |  | 57.0(31.6-80.8) | 62.8(37.5-82.3) | 0.028 |

**Table S5.** **Hazard Ratios and 95% CIs of Cardiovascular Outcomes According to the Tertile of Time in Target Range for Serum Potassium stratify by Spironolactone Treatment**

| TTR | Drug group | Events (No.) | Follow-up Duration  (Person-Year) | Incident Rate  (Per 1000 Person-Year) | Unadjusted  *HR (95% CI) | P for interaction |
| --- | --- | --- | --- | --- | --- | --- |
| Primary outcome: Death from Cardiovascular Causes, Aborted Cardiac Arrest, or Hospitalization for The Management of Heart Failure | | | | | |  |
| 0% to 34.0% | Placebo | 142/633 | 1927 | 73.69(62.52-86.87) | 0.969(0.823-1.141) | 0.524 |
|  | Spironolactone | 79/414 | 1241 | 63.64(51.04-79.34) | 1.053(0.836-1.327) |  |
| 34.1% to 67.0% | Placebo | 72/473 | 1756 | 41.00(32.54-51.65) | 1.155(0.910-1.466) | 0.259 |
|  | Spironolactone | 99/575 | 2026 | 48.86(10.12-59.49) | 0.972(0.800-1.181) |  |
| 67.1% to 100% | Placebo | 59/460 | 1520 | 38.83(30.08-50.11) | 1.085(0.831-1.416) | 0.673 |
|  | Spironolactone | 73/586 | 2018 | 36.17(28.76-45.50) | 1.036(0.818-1.311) |  |
| Secondary outcome: All-Cause Mortality | | | | | |  |
| 0% to 34.0% | Placebo | 106/633 | 2109 | 50.27(41.56-60.81) | 1.040(0.863-1.253) | 0.748 |
|  | Spironolactone | 79/414 | 1315 | 60.10(48.20-74.92) | 0.984(0.780-1.241) |  |
| 34.1% to 67.0% | Placebo | 64/473 | 1831 | 34.96(27.36-44.66) | 1.164(0.903-1.500) | 0.029 |
|  | Spironolactone | 82/575 | 2110 | 38.86(31.29-48.24) | 0.811(0.655-1.004) |  |
| 67.1% to 100% | Placebo | 50/460 | 1588 | 31.48(23.86-41.54) | 1.124(0.836-1.511) | 0.763 |
|  | Spironolactone | 56/586 | 2096 | 26.72(20.56-34.71) | 1.063(0.811-1.394) |  |

Abbreviations: TTR, time in target range; SD, standard deviation; HR, hazard ratio; CI, confidence interval.

**Table S6. Association of Time in Target Range for Serum Potassium with Cardiovascular Outcomes Adjusted for Number of Anti-Heart Failure Medication**

| TTR | Events (No.) | Follow-up Duration  (Person-Year) | Incident Rate  (Per 1000 Person-Year) | Model *  HR (95% CI) |
| --- | --- | --- | --- | --- |
| Primary outcome: Death from Cardiovascular Causes, Aborted Cardiac Arrest, or Hospitalization for The Management of Heart Failure | | | | |
| 0% to 34% | 221 | 3168 | 69.75(61.14-79.58) | 1(ref.) |
| 34.1% to 67% | 171 | 3783 | 45.21(38.92-52.52) | 0.739(0.602-0.907) |
| 67.1% to 100% | 131 | 3538 | 37.31(31.46-44.25) | 0.716(0.572-0.895) |
| *P* for trend |  |  |  | 0.003 |
| Secondary outcome: All-Cause Mortality | | | | |
| 0% to 34.0% | 185 | 3423 | 54.04(46.79-62.42) | 1(ref.) |
| 34.1% to 67.0% | 146 | 3941 | 37.04(31.50-43.57) | 0.748(0.599-0.934) |
| 67.1% to 100% | 106 | 3685 | 28.77(23.78-34.80) | 0.649(0.507-0.831) |
| *P* for trend |  |  |  | ＜0.001 |

Abbreviations: TTR, time in target range; SD, standard deviation; HR, hazard ratio; CI, confidence interval; BMI, body mass index; eGFR, estimated glomerular filtration rate; NYHA, New York Heart Association; LVEF, left ventricular ejection fraction; CVD, cardiovascular disease.

Primary outcome: death from cardiovascular causes, aborted cardiac arrest, or Hospitalization for the management of heart failure; Secondary outcome: all-cause mortality.

*Adjusted for baseline demographics (age and sex), BMI, region, race, eGFR, heart rate, NYHA class, LVEF, hypertension, diabetes mellitus, CVD, beta-blocker use, diuretic use, and number of anti-heart failure medications.

**Table S7. Sensitivity Analysis: Unadjusted and Adjusted Associations of Serum Potassium Time in Target Range (4.3–4.6) and Cardiovascular Outcomes**

| TTR  Z score | Events (No.) | Follow-up Duration  (Person-Year) | Incident Rate  (Per 1000 Person-Year) | Unadjusted  HR* (95% CI) | Model 1  HR* (95% CI) | Model 2  HR* (95% CI) | Model 3  HR* (95% CI) |
| --- | --- | --- | --- | --- | --- | --- | --- |
| Primary outcome | 524 | 10489 | 49.96(45.86-54.43) | 0.917(0.834-1.008) | 0.892(0.811-0.980) | 0.902(0.822-0.990) | 0.900(0.819-0.988) |
| Secondary outcome | 437 | 11049 | 39.55(36.01-43.44) | 0.904(0.814-1.004) | 0.878(0.791-0.975) | 0.887(0.800-0.984) | 0.885(0.799-0.982) |

*HR per 1-SD increase in time in target range.

Abbreviations: TTR, time in target range; SD, standard deviation; HR, hazard ratio; CI, confidence interval; BMI, body mass index; eGFR, estimated glomerular filtration rate; NYHA, New York Heart Association; LVEF, left ventricular ejection fraction; CVD, cardiovascular disease.

Primary outcome: death from cardiovascular causes, aborted cardiac arrest, or hospitalization for the management of heart failure; Secondary outcome: all-cause mortality.

Model 1, Adjusted for baseline demographics (age and sex) and BMI.

Model 2, Adjusted for baseline demographics (age and sex), BMI, and region, race, eGFR, heart rate, NYHA class, LVEF, hypertension, diabetes mellitus, and CVD.

Model 3, Adjusted for baseline demographics (age and sex), BMI, region, race, eGFR, heart rate, NYHA class, LVEF, hypertension, diabetes mellitus, CVD, and use of beta-blockers and diuretics.

**Table S8. Sensitivity Analysis: Unadjusted and Adjusted Associations of Serum Potassium Time in Target Range (4.6–4.9) and Cardiovascular Outcomes.**

| TTR  Z score | Events (No.) | Follow-up Duration  (Person-Year) | Incident Rate  (Per 1000 Person-Year) | Unadjusted  HR* (95% CI) | Model 1  HR* (95% CI) | Model 2  HR* (95% CI) | Model 3  HR* (95% CI) |
| --- | --- | --- | --- | --- | --- | --- | --- |
| Primary outcome | 524 | 10489 | 49.96(45.86-54.43) | 0.764(0.696-0.839) | 0.807(0.734-0.887) | 0.918(0.831-1.013) | 0.918(0.832-1.014) |
| Secondary outcome | 437 | 11049 | 39.55(36.01-43.44) | 0.765(0.690-0.848) | 0.796(0.717-0.884) | 0.877(0.787-0.987) | 0.879(0.788-0.980) |

*HR per 1-SD increase in time in target range.

Abbreviations: TTR, time in target range; SD, standard deviation; HR, hazard ratio; CI, confidence interval; BMI, body mass index; eGFR, estimated glomerular filtration rate; NYHA, New York Heart Association; LVEF, left ventricular ejection fraction; CVD, cardiovascular disease.

Primary outcome: death from cardiovascular causes, aborted cardiac arrest, or hospitalization for the management of heart failure; Secondary outcome: all-cause mortality.

Model 1, Adjusted for baseline demographics (age and sex) and BMI.

Model 2, Adjusted for baseline demographics (age and sex), BMI, and region, race, eGFR, heart rate, NYHA class, LVEF, hypertension, diabetes mellitus, and CVD.

Model 3, Adjusted for baseline demographics (age and sex), BMI, region, race, eGFR, heart rate, NYHA class, LVEF, hypertension, diabetes mellitus, CVD, and use of beta-blockers and diuretics.

**Table S9. Sensitivity Analysis: Unadjusted and Adjusted Associations of Serum Potassium Time in Target Range (4.0–4.3) and Cardiovascular Outcomes**

| TTR  Z score | Events (No.) | Follow-up Duration  (Person-Year) | Incident Rate  (Per 1000 Person-Year) | Unadjusted  HR* (95% CI) | Model 1  HR* (95% CI) | Model 2  HR* (95% CI) | Model 3  HR* (95% CI) |
| --- | --- | --- | --- | --- | --- | --- | --- |
| Primary outcome | 524 | 10489 | 49.96(45.86-54.43) | 1.185(1.090-1.289) | 1.125(1.033-1.224) | 1.051(0.962-1.149) | 1.047(0.958-1.144) |
| Secondary outcome | 437 | 11049 | 39.55(36.01-43.44) | 1.235(1.128-1.352) | 1.174(1.071-1.287) | 1.113(1.012-1.225) | 1.111(1.009-1.222) |

*HR per 1-SD increase in time in target range.

Abbreviations: TTR, time in target range; SD, standard deviation; HR, hazard ratio; CI, confidence interval; BMI, body mass index; eGFR, estimated glomerular filtration rate; NYHA, New York Heart Association; LVEF, left ventricular ejection fraction; CVD, cardiovascular disease.

Primary outcome: death from cardiovascular causes, aborted cardiac arrest, or hospitalization for the management of heart failure; Secondary outcome: all-cause mortality.

Model 1, Adjusted for baseline demographics (age and sex) and BMI.

Model 2, Adjusted for baseline demographics (age and sex), BMI, region, race, eGFR, heart rate, NYHA class, LVEF, hypertension, diabetes mellitus, and CVD.

Model 3, Adjusted for baseline demographics (age and sex), BMI, region, race, eGFR, heart rate, NYHA class, LVEF, hypertension, diabetes mellitus, CVD, and use of beta-blockers and diuretics.

**Table S10. Sensitivity Analysis: Unadjusted and Adjusted Associations of Serum Potassium Time in Target Range (4.9–5.2) and Cardiovascular Outcomes**

| TTR  Z score | Events (No.) | Follow-up Duration  (Person-Year) | Incident Rate  (Per 1000 Person-Year) | Unadjusted  HR* (95% CI) | Model 1  HR* (95% CI) | Model 2  HR* (95% CI) | Model 3  HR* (95% CI) |
| --- | --- | --- | --- | --- | --- | --- | --- |
| Primary outcome | 524 | 10489 | 49.96(45.86-54.43) | 0.948(0.865-1.039) | 0.967(0.882-1.061) | 1.018(0.928-1.117) | 1.025(0.934-1.124) |
| Secondary outcome | 437 | 11049 | 39.55(36.01-43.44) | 0.937(0.846-1.037) | 0.952(0.860-1.055) | 0.989(0.892-1.097) | 0.994(0.896-1.102) |

*HR per 1-SD increase in time in target range.

Abbreviations: TTR, time in target range; SD, standard deviation; HR, hazard ratio; CI, confidence interval; BMI, body mass index; eGFR, estimated glomerular filtration rate; NYHA, New York Heart Association; LVEF, left ventricular ejection fraction; CVD, cardiovascular disease.

Primary outcome: death from cardiovascular causes, aborted cardiac arrest, or hospitalization for the management of heart failure; Secondary outcome: all-cause mortality.

Model 1, Adjusted for baseline demographics (age and sex) and BMI.

Model 2, Adjusted for baseline demographics (age and sex), BMI, and region, race, eGFR, heart rate, NYHA class, LVEF, hypertension, diabetes mellitus, and CVD.

Model 3, Adjusted for baseline demographics (age and sex), BMI, region, race, eGFR, heart rate, NYHA class, LVEF, hypertension, diabetes mellitus, CVD, and use of beta-blockers and diuretics.

**Table S11. Hazard Ratios and 95% CIs of Cardiovascular Outcomes According to the Tertile of Time in Target Range for Serum Potassium by Using ≥ 3 Serum Potassium Measurements**

| TTR | Events (No.) | Follow-up Duration  (Person-Year) | Incident Rate  (Per 1000 Person-Year) | Unadjusted  HR (95% CI) | Model 1  HR (95% CI) | Model 2  HR (95% CI) | Model 3  HR (95% CI) |
| --- | --- | --- | --- | --- | --- | --- | --- |
| Primary outcome: Death from Cardiovascular Causes, Aborted Cardiac Arrest, or Hospitalization for The Management of Heart Failure | | | | | | | |
| 0% to 34.0% | 247 | 3197 | 77.26(68.20-87.52) | 1(ref.) | 1(ref.) | 1(ref.) | 1(ref.) |
| 34.1% to 67.0% | 196 | 3863 | 50.74(44.11-58.37) | 0.677(0.561-0.816) | 0.685(0.568-0.827) | 0.765(0.632-0.925) | 0.763(0.631-0.922) |
| 67.1% to 100% | 153 | 3599 | 42.51(36.28-49.81) | 0.561(0.459-0.687) | 0.603(0.492-0.739) | 0.752(0.611-0.925) | 0.749(0.609-0.922) |
| *P* for trend |  |  |  | < 0.001 | < 0.001 | 0.004 | 0.004 |
| Per SD increase | 596 | 10659 | 55.92(51.60-60.59) | 0.794(0.731-0.863) | 0.816(0.750-0.888) | 0.899(0.824-0.981) | 0.898(0.823-0.980) |
| Secondary outcome: All-Cause Mortality | | | | | | | |
| 0% to 34.0% | 191 | 3506 | 54.47(47.27-62.77) | 1(ref.) | 1(ref.) | 1(ref.) | 1(ref.) |
| 34.1% to 67.0% | 165 | 4070 | 40.54(34.80-47.22) | 0.729(0.591-0.898) | 0.750(0.608-0.924) | 0.806(0.652-0.995) | 0.807(0.653-0.997) |
| 67.1% to 100% | 124 | 3766 | 32.92(27.61-39.26) | 0.599(0.478-0.751) | 0.613(0.488-0.770) | 0.729(0.577-0.921) | 0.728(0.576-0.920) |
| *P* for trend |  |  |  | < 0.001 | < 0.001 | 0.006 | 0.006 |
| Per SD increase | 480 | 11342 | 42.32(38.70-46.28) | 0.818(0.745-0.898) | 0.827(0.752-0.909) | 0.889(0.806-0.980) | 0.889(0.806-0.980) |

Primary outcome: death from cardiovascular causes, aborted cardiac arrest, or hospitalization for the management of heart failure; Secondary outcome: all-cause mortality.

Model 1, Adjusted for baseline demographics (age and sex) and BMI.

Model 2, Adjusted for Model 1 + region, race, eGFR, heart rate, New York Heart Association class, left ventricular ejection fraction, hypertension, diabetes mellitus, and CVD.

Model 3, Adjusted for Model 2 + use of beta-blockers and diuretics.

Abbreviations: TTR, time in target range, HR, hazard ratio; CI, confidence interval; BMI, body mass index; eGFR, estimated glomerular filtration rate; CVD, cardiovascular disease, SD, standard deviation.

**Table S12 Hazard Ratios and 95% CIs of Aborted Cardiac Arrest or Ventricular Tachycardia Event According to Mean, Variability and TTR for Serum Potassium**

| **TTR** | **Events (No.)** | **Follow-up Duration**  **(Person-Year)** | **Incident Rate**  **(Per 1000 Person-Year)** | **Unadjusted**  **HR (95% CI)** |
| --- | --- | --- | --- | --- |
| **Mean serum potassium** |  |  |  |  |
| Tertile 1 | 14 | 3494 | 4.01(2.37-6.76) | 1(ref.) |
| Tertile 2 | 4 | 3815 | 1.05(0.39-2.79) | 0.263(0.086-0.798) |
| Tertile 3 | 5 | 3719 | 1.34(0.56-3.23) | 0.338(0.122-0.939) |
| *P* for trend |  |  |  | 0.019 |
| Per SD increase | 23 | 11029 | 2.09(1.39-3.14) | 0.589(0.300-1.157) |
| **Serum potassium SD** |  |  |  |  |
| Tertile 1 | 2 | 3349 | 0.597(0.149-2.388) | 1(ref.) |
| Tertile 2 | 10 | 3821 | 2.62(1.41-4.86) | 4.440(0.972-20.291) |
| Tertile 3 | 11 | 3859 | 2.85(1.58-5.15) | 4.858(1.075-21.947) |
| *P* for trend |  |  |  | 0.043 |
| Per SD increase | 23 | 11029 | 2.09(1.39-3.14) | 0.988(0.641-1.522) |
| **Serum potassium time in target range** |  |  |  |  |
| 0% to 34.0% | 12 | 3412 | 3.52(2.00-6.19) | 1(ref.) |
| 34.1% to 67.0% | 7 | 3933 | 1.78(0.85-3.73) | 0.509(0.200-1.297) |
| 67.1% to 100% | 4 | 3684 | 1.09(0.41-2.89) | 0.311(0.100-0.964) |
| *P* for trend |  |  |  | 0.031 |
| Per SD increase | 23 | 11029 | 2.09(1.39-3.14) | 0.636(0.413-0.980) |

Abbreviations: TTR, time in target range; SD, standard deviation; HR, hazard ratio; CI, confidence interval.

**Figure S1. Selection of Study Population**


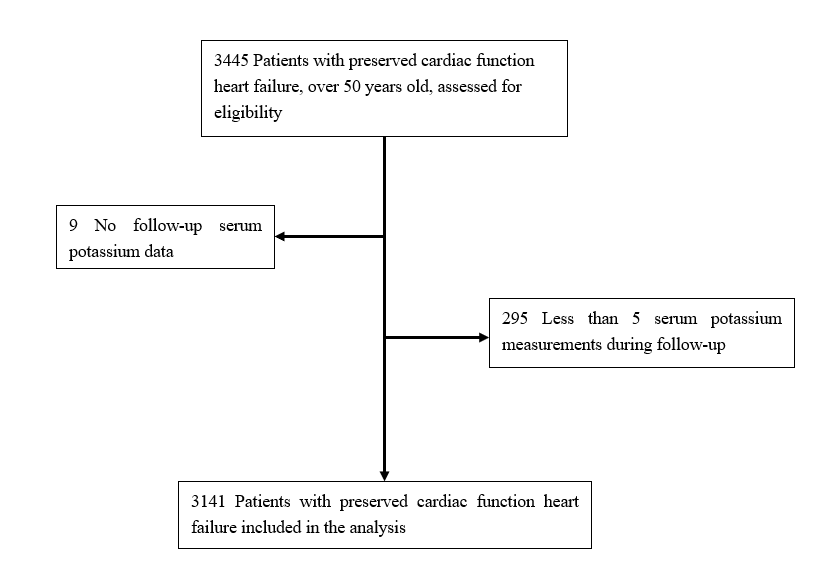


**Figure S2. Subgroup Analysis:** **The Association Between Serum Potassium Time in Target Range and Cardiovascular Outcomes According to Age**


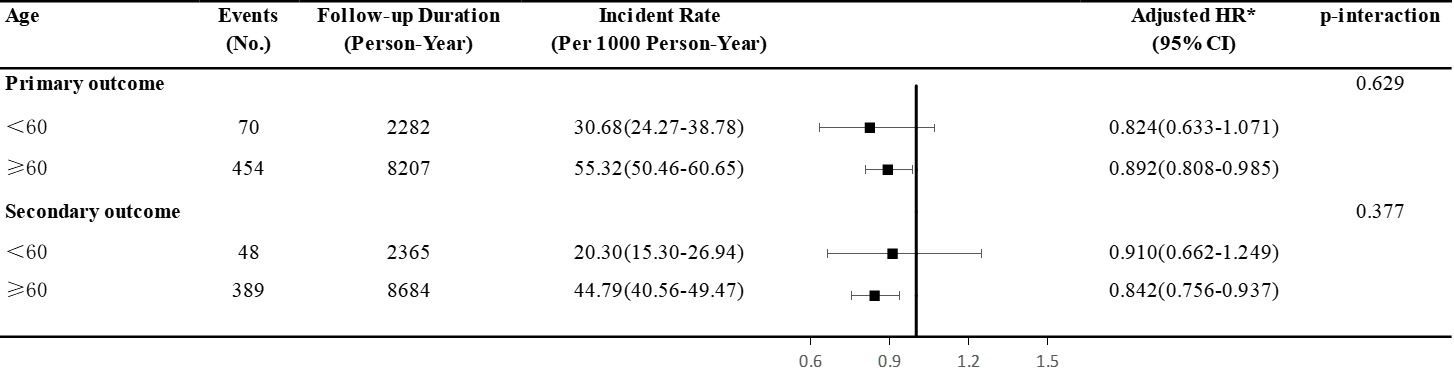


*HR per 1-SD increase in TTR.

Primary outcome: death from cardiovascular causes, aborted cardiac arrest, or hospitalization for the management of heart failure; Secondary outcome: all-cause mortality.

Adjusted for baseline demographics (age and sex), BMI, region, race, eGFR, heart rate, NYHA class, LVEF, hypertension, diabetes mellitus, CVD, and use of beta-blockers and diuretics.

Abbreviations: TTR, time in target range; SD, standard deviation; HR, hazard ratio; CI, confidence interval; BMI, body mass index; eGFR, estimated glomerular filtration rate; NYHA, New York Heart Association; LVEF, left ventricular ejection fraction; CVD, cardiovascular disease.

**Figure S3. Subgroup Analysis:** **The Association Between Serum Potassium Time in Target Range and Cardiovascular Outcomes According to Region**

*HR per 1-SD increase in TTR.

Primary outcome: death from cardiovascular causes, aborted cardiac arrest, or hospitalization for the management of heart failure; Secondary outcome: all-cause mortality.

Adjusted for baseline demographics (age and sex), BMI, region, race, eGFR, heart rate, NYHA class, LVEF, hypertension, diabetes mellitus, CVD, and use of beta-blockers and diuretics.

Abbreviations: TTR, time in target range; SD, standard deviation; HR, hazard ratio; CI, confidence interval; BMI, body mass index; eGFR, estimated glomerular filtration rate; NYHA, New York Heart Association; LVEF, left ventricular ejection fraction; CVD, cardiovascular disease.

**Figure S4. Subgroup Analysis: The Association Between Serum Potassium Time in Target Range and Cardiovascular Outcomes According to Sex**

*HR per 1-SD increase in TTR.

Primary outcome: death from cardiovascular causes, aborted cardiac arrest, or hospitalization for the management of heart failure; Secondary outcome: all-cause mortality.

Adjusted for baseline demographics (age and sex), BMI, region, race, eGFR, heart rate, NYHA class, LVEF, hypertension, diabetes mellitus, CVD, and use of beta-blockers and diuretics.

Abbreviations: TTR, time in target range; SD, standard deviation; HR, hazard ratio; CI, confidence interval; BMI, body mass index; eGFR, estimated glomerular filtration rate; NYHA, New York Heart Association; LVEF, left ventricular ejection fraction; CVD, cardiovascular disease.

**Figure S5. Subgroup Analysis: The Association Between Serum Potassium Time in Target Range and Cardiovascular Outcomes According to BMI**

*HR per 1-SD increase in TTR.

Primary outcome: death from cardiovascular causes, aborted cardiac arrest, or hospitalization for the management of heart failure; Secondary outcome: all-cause mortality.

Adjusted for baseline demographics (age and sex), BMI, region, race, eGFR, heart rate, NYHA class, LVEF, hypertension, diabetes mellitus, CVD, and use of beta-blockers and diuretics.

Abbreviations: TTR, time in target range; SD, standard deviation; HR, hazard ratio; CI, confidence interval; BMI, body mass index; eGFR, estimated glomerular filtration rate; NYHA, New York Heart Association; LVEF, left ventricular ejection fraction; CVD, cardiovascular disease.

**Figure S6. Subgroup Analysis: The Association Between Serum Potassium Time in Target Range and Cardiovascular Outcomes According to NYHA class**

*HR per 1-SD increase in TTR.

Primary outcome: death from cardiovascular causes, aborted cardiac arrest, or hospitalization for the management of heart failure; Secondary outcome: all-cause mortality.

Adjusted for baseline demographics (age and sex), BMI, region, race, eGFR, heart rate, NYHA class, LVEF, hypertension, diabetes mellitus, CVD, and use of beta-blockers and diuretics.

Abbreviations: TTR, time in target range; SD, standard deviation; HR, hazard ratio; CI, confidence interval; BMI, body mass index; eGFR, estimated glomerular filtration rate; NYHA, New York Heart Association; LVEF, left ventricular ejection fraction; CVD, cardiovascular disease.

**Figure S7. Subgroup Analysis: The Association Between Serum Potassium Time in Target Range and Cardiovascular Outcomes According to eGFR**

*HR per 1-SD increase in TTR.

Primary outcome: death from cardiovascular causes, aborted cardiac arrest, or hospitalization for the management of heart failure; Secondary outcome: all-cause mortality.

Adjusted for baseline demographics (age and sex), BMI, region, race, eGFR, heart rate, NYHA class, LVEF, hypertension, diabetes mellitus, CVD, and use of beta-blockers and diuretics.

Abbreviations: TTR, time in target range; SD, standard deviation; HR, hazard ratio; CI, confidence interval; BMI, body mass index; eGFR, estimated glomerular filtration rate; NYHA, New York Heart Association; LVEF, left ventricular ejection fraction; CVD, cardiovascular disease.

**Figure S8. Subgroup Analysis: The Association Between Serum Potassium Time in Target Range and Cardiovascular Outcomes According to AF**

*HR per 1-SD increase in TTR.

Primary outcome: death from cardiovascular causes, aborted cardiac arrest, or hospitalization for the management of heart failure; Secondary outcome: all-cause mortality.

Adjusted for baseline demographics (age and sex), BMI, region, race, eGFR, heart rate, NYHA class, LVEF, hypertension, diabetes mellitus, CVD, and use of beta-blockers and diuretics.

Abbreviations: AF, atrial fibrillation; TTR, time in target range; SD, standard deviation; HR, hazard ratio; CI, confidence interval; BMI, body mass index; eGFR, estimated glomerular filtration rate; NYHA, New York Heart Association; LVEF, left ventricular ejection fraction; CVD, cardiovascular disease.

**Figure S9. Subgroup Analysis: The Association Between Serum Potassium Time in Target Range and Cardiovascular Outcomes According to the Use of Spironolactone**

*HR per 1-SD increase in TTR.

Primary outcome: death from cardiovascular causes, aborted cardiac arrest, or hospitalization for the management of heart failure; Secondary outcome: all-cause mortality.

Adjusted for baseline demographics (age and sex), BMI, region, race, eGFR, heart rate, NYHA class, LVEF, hypertension, diabetes mellitus, CVD, and use of beta-blockers and diuretics.

Abbreviations: TTR, time in target range; SD, standard deviation; HR, hazard ratio; CI, confidence interval; BMI, body mass index; eGFR, estimated glomerular filtration rate; NYHA, New York Heart Association; LVEF, left ventricular ejection fraction; CVD, cardiovascular disease.

**Figure S10. Subgroup Analysis: The Association Between Serum Potassium Time in Target Range and Cardiovascular Outcomes According to Race**

*HR per 1-SD increase in TTR.

Primary outcome: death from cardiovascular causes, aborted cardiac arrest, or hospitalization for the management of heart failure; Secondary outcome: all-cause mortality.

Adjusted for baseline demographics (age and sex), BMI, region, race, eGFR, heart rate, NYHA class, LVEF, hypertension, diabetes mellitus, CVD, and use of beta-blockers and diuretics.

Abbreviations: TTR, time in target range; SD, standard deviation; HR, hazard ratio; CI, confidence interval; BMI, body mass index; eGFR, estimated glomerular filtration rate; NYHA, New York Heart Association; LVEF, left ventricular ejection fraction; CVD, cardiovascular disease.
