# Supplementary material for: Association of serum potassium time in target range with cardiovascular outcomes in patients with HFpEF
Source: Open Heart. 2025 Aug 21;12(2):e003439. doi: 10.1136/openhrt-2025-003439 (PMC12410661; doi:10.1136/openhrt-2025-003439)
Supplement: online supplemental file 2 [file openhrt-12-2-s002.pdf]

# Serum Potassium Time in Target Range as an Independent Predictor of Cardiovascular Outcomes in Heart Failure with Preserved Ejection Fraction

**3,141 Patients with preserved cardiac function heart failure included in the analysis**

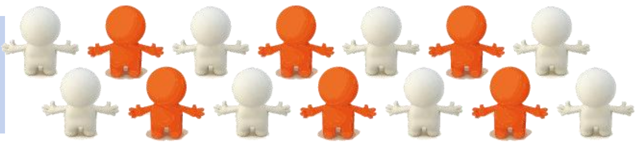

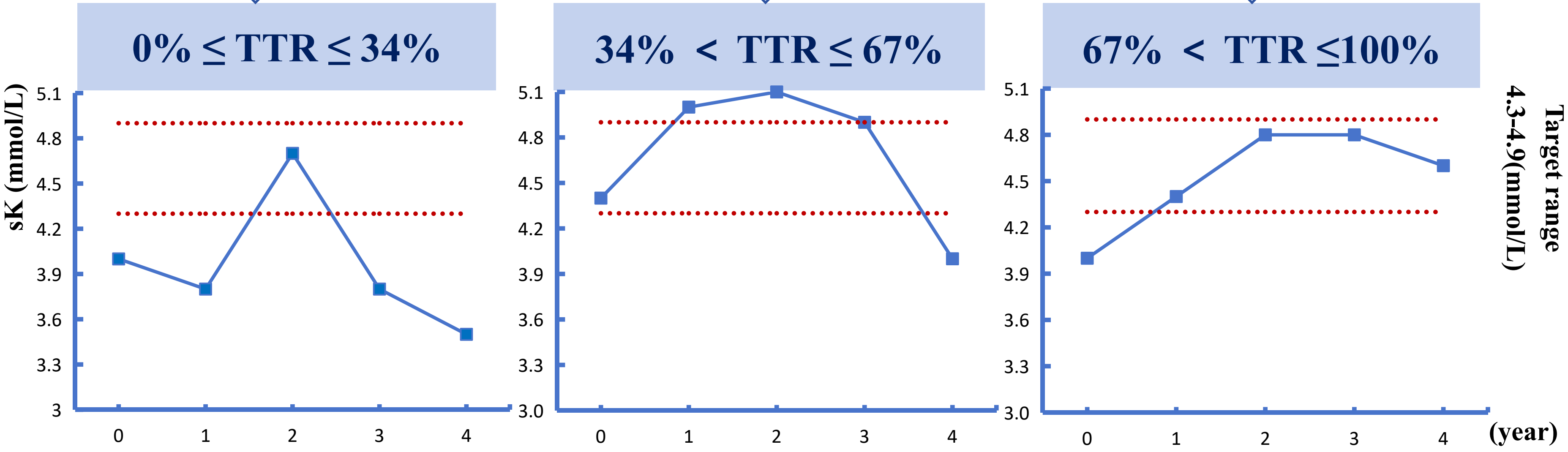


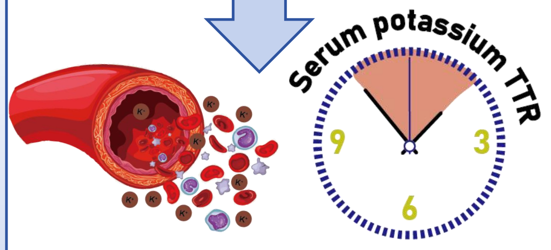


**Higher Time-in-Target Range**

**Primary outcome: Death from Cardiovascular Causes, Aborted Cardiac Arrest, or Hospitalization for The Management of Heart Failure**

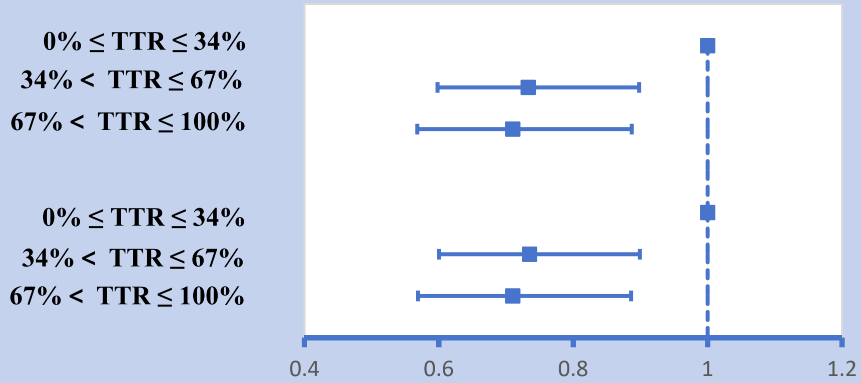


| | |
|---|---|
| 1 | |
| 0.733(0.598-0.898) | Higher sK TTR is associated with a decreased risk of primary outcome, independent of mean sK |
| 0.710(0.568-0.887) | |
| 1 | |
| 0.735(0.600-0.899) | Higher sK TTR is associated with a decreased risk of primary outcome, independent of sK Variability |
| 0.710(0.569-0.886) | |

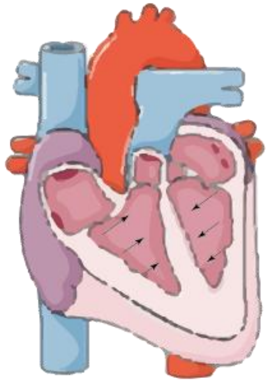

**Lower Risk of Primary outcome**
